# Ethanol injection into the Marshall vein provoking a pericardial effusion resulting in a fatal complication in a patient with persistent atrial fibrillation

**DOI:** 10.1002/ccr3.1076

**Published:** 2017-07-30

**Authors:** Kazuo Kato, Osamu Igawa, Shin‐ichiro Morimoto, Ryosuke Kametani, Akimitsu Tanaka, Hideo Hattori

**Affiliations:** ^1^ Department of Cardiology Nagoya Tokushukai General Hospital Kasugai Japan; ^2^ Department of Internal Medicine Nihon Medical University Tokyo Japan; ^3^ Department of Cardiology Fujita Health University Toyoake Japan

**Keywords:** Atrial fibrillation, cardiac tamponade, chemical ablation, complication, ethanol, vein of Marshall

## Abstract

An EIM (ethanol infusion into the vein of Marshall [VOM]) provoked a fatal complication in a chronic hemodialysis patient. Autopsy revealed a lacerated VOM covered with thrombi as the only potential cause. The EIM caused vascular damage and clots resulting in myocardial necrosis and interstitial bleeding around the lacerated VOM.

## Introduction

The vein of Marshall (VOM) is thought to be one of the therapeutic targets for arrhythmias, especially atrial fibrillation (AF) 1, 2, 3. Recently, ethanol infusions into the VOM (EIM) have been reported as an alternative option for left atrial ablation 4, 5, 6. This new method has been described as being safe and effective because no serious complications have been reported 1, 2, 3, 4, 5, 6. We experienced, however, a case that developed nonocclusive mesenteric ischemia (NOMI) following transient hypotension due to a hemopericardium 2 h after an EIM, which led to multiple systemic organ failure and death 2 days after the procedure. A postmortem examination revealed a lacerated VOM and no other procedural related injuries. To the best of our knowledge, this is the first report to histopathologically exhibit an EIM lesion during the acute phase.

## Case Report

A 57‐year‐old woman, on hemodialysis for 20 years, was referred to our hospital to treat drug‐resistant AF in April 2015. She suffered from paroxysmal AF since 2009, and underwent two catheter ablation procedures for daily episodes of AF in January 2012 and March 2013. Pulmonary vein (PV) isolation and the creation of mitral isthmus (MI) and cavo‐tricuspid isthmus block were performed during the first session, and reconnections of the former ablation lines were targeted during the second session. After those sessions, she was relieved from her AF burden for 6 months. However, AF recurred and exacerbated her hemodynamic condition leading to intolerance of hemodialysis from January 2015, so she decided to undergo a third session.

## Ablation Procedure

**Figure 1:**
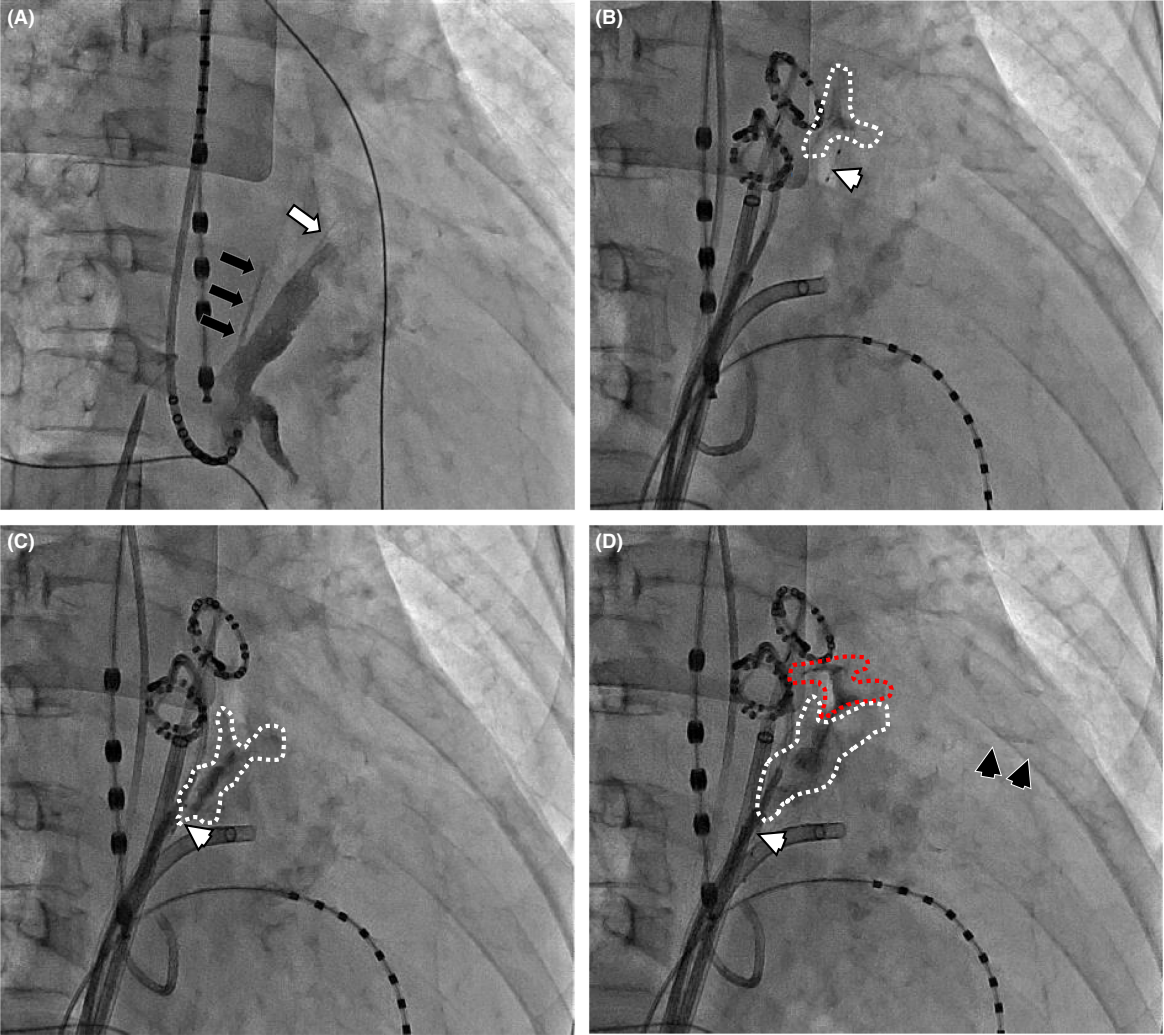
Right anterior oblique projection view of the procedural series of the ethanol infusion into the vein of Marshall (VOM). (A) Retrograde subselective venogram of the VOM. The white arrow indicates the distal VOM and the black arrows indicate the proximal VOM. Both of those extend upward along the anterior ridge of the left atrial appendage (LAA). (B) Cannulation of an angioplasty wire and balloon (white arrowhead) into the distal VOM, and an ethanol infusion following a contrast medium injection. The area surrounded by the white dashed lines indicates the area stained by the contrast medium injection. (C) The first ethanol infusion into the proximal VOM performed in the same manner as above. (D) The third ethanol infusion into the proximal VOM. The area surrounded by the red dashed lines indicates the leakage from the VOM into the pericardial space. Note that the pericardial space is also stained, which is not recognized in (A) through (C) (black arrowheads).

After informed consent was obtained for all procedures including the EIM, which was approved by the review board of this hospital, catheter ablation was performed. Her left atrial diameter was 51 mm and left ventricular ejection fraction 56% the day before the ablation procedure, and sinus rhythm was maintained at the beginning of the procedure. Her esophageal temperature was monitored (Sensi‐Therm™, St. Jude Medical, St. Paul, MN), and CS venography was performed using the infusion port of a duodecapolar CS electrode catheter (CSL™, St. Jude Medical) visibly confirming two oblique veins of the left atrium (LA) (i.e., vein of Marshall [VOM]) (Fig. 1A). Then, a standard trans‐septal puncture (one puncture with three sheaths in the LA) and creation of an LA geometry voltage map using a circular catheter and ablation catheter with the EnSite NavX 3D mapping system (St. Jude Medical) were performed. Reconnections of the left superior PV (LSPV), left inferior PV (LIPV), and right superior PV (RSPV) were present.

After the duodecapolar circular mapping, catheters were placed into the LSPV and LIPV to record their potentials, and an EIM procedure was performed as described below before performing a conventional catheter ablation as previously described 4. An angiographic catheter for the left internal mammary artery (6 Fr IM™, Asahi Intec, Tokyo, Japan) was inserted into coronary sinus close to the ostium of the VOM. Through that catheter, a guidewire (0.014″ Cruise™ for peripheral angioplasty, Asahi Intec) and balloon catheter (8 mm length Apex OTW™ with a 2‐mm nominal diameter for angioplasty, Boston Scientific, Boston, MA) were advanced as far as possible into the VOM. Then, following an injection of 0.2‐0.3 mL of radiographic contrast medium, 2.0 mL of dehydrated ethanol was injected once into the distal VOM and then three times from just distal to the ostium into the proximal VOM over 2 min each after selective VOM venography through the lumen of the angioplasty balloon (Fig. 1B and C).

**Figure 2:**
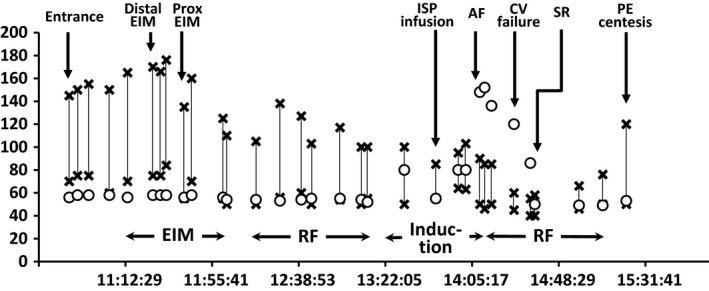
Time series of her blood pressure (BP) and heart rate (HR) corresponding to each procedure. The X mark and open circle indicate the BP and HR, respectively. The BP and HR were stably maintained until performing the ethanol infusion into the vein of Marshall (EIM). The BP decreased after the EIM, and the systolic BP was maintained at more than 100 mmHg throughout the procedure. However, burst stimulation with isoproterenol (ISP) provoked a refractory atrial tachycardia and atrial fibrillation (AF), leading to hypotension that lasted until sinus rhythm was restored by additional RF ablation, and a pericardiocentesis was performed. CV indicates cardioversion; RF, radiofrequency ablation; and PE, pericardial effusion.

During the third ethanol injection into the proximal VOM, contrast medium leaked out through the VOM and enhanced the pericardial space (Fig. 1D). Echocardiography revealed trivial pericardial effusion, and her blood pressure (BP) decreased transiently but normalized soon after without centesis (Fig. 2). Therefore, the echocardiography was frequently checked in addition to invasive BP monitoring to see whether the pericardial effusion had increased, and proceeded with a conventional radiofrequency (RF) ablation. RF energy was applied to the PV antrum, where an LA‐PV connection had recovered, LA roof, and cavo‐tricuspid isthmus, and bidirectional conduction block of all those lesions was verified.

Until then, her hemodynamic condition was stable and the pericardial effusion on echocardiography had not increased, so LA burst stimulation up to 300 ppm was applied under an isoproterenol administration at 20 mcg/min to check whether any atrial arrhythmia could be induced, which unfortunately provoked AF refractory to cardioversion with a rapid ventricular response and her BP decreased. The echocardiography revealed no significant increase in the pericardial effusion and an insufficient pericardial space for a safe centesis even after her BP had decreased. Therefore, it was decided to proceed with additional RF ablation to terminate the cardioversion refractory AF because her BP was kept at >60 mmHg without any vasopressors and had not collapsed. An RF application to the MI finally restored her rhythm, and a pericardiocentesis was performed draining 225 mL from the hemopericardium, because the pericardial effusion had slightly increased after several cardioversion attempts at 150 joules.

Her hemodynamic condition was completely restored, and no rebleeding was documented under frequent ultrasound monitoring; however, hypoglycemia, metabolic acidosis, and an elevation of her liver transaminase level gradually developed after the procedure, and she suddenly collapsed 15 h later. An abdominal computed tomography (CT) revealed remarkable distention and fluid retention of the intestines, especially of the colon, without any occlusive lesions, suggesting paresis of the intestines had occurred due to NOMI. Her condition steadily deteriorated, and she died 2 days after the procedure.

**Figure 3:**
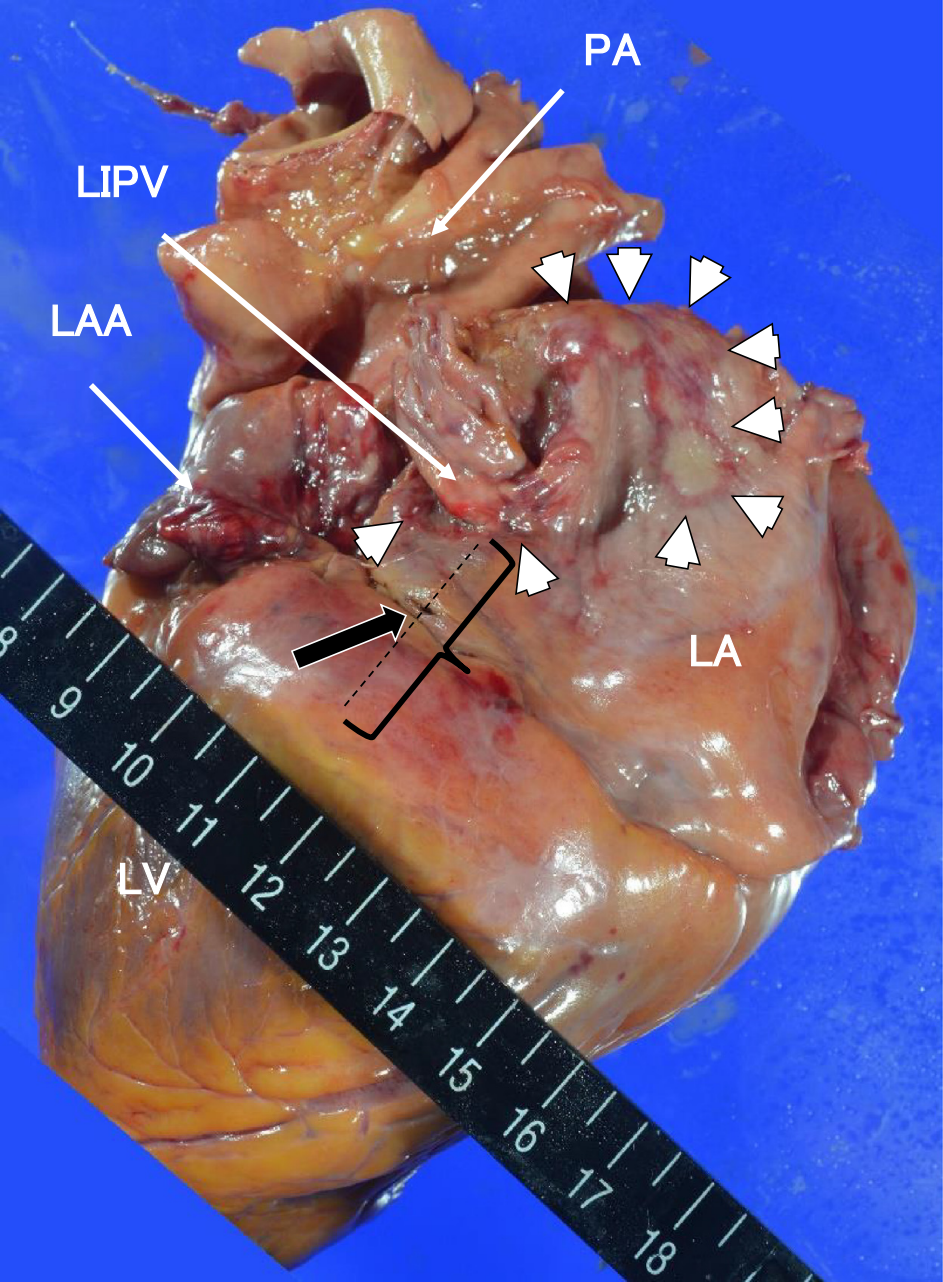
Gross postmortem examination of the heart. Posterior view of the extracted heart. There are visible transmural lesions on the roof, posterior left atrium, and bottom of the left inferior pulmonary vein (LIPV) (white arrowheads). A laceration of the vein of Marshall (VOM) covered with thrombi (removed) is seen between the atrioventricular groove and LIPV (black arrow).

**Figure 4:**
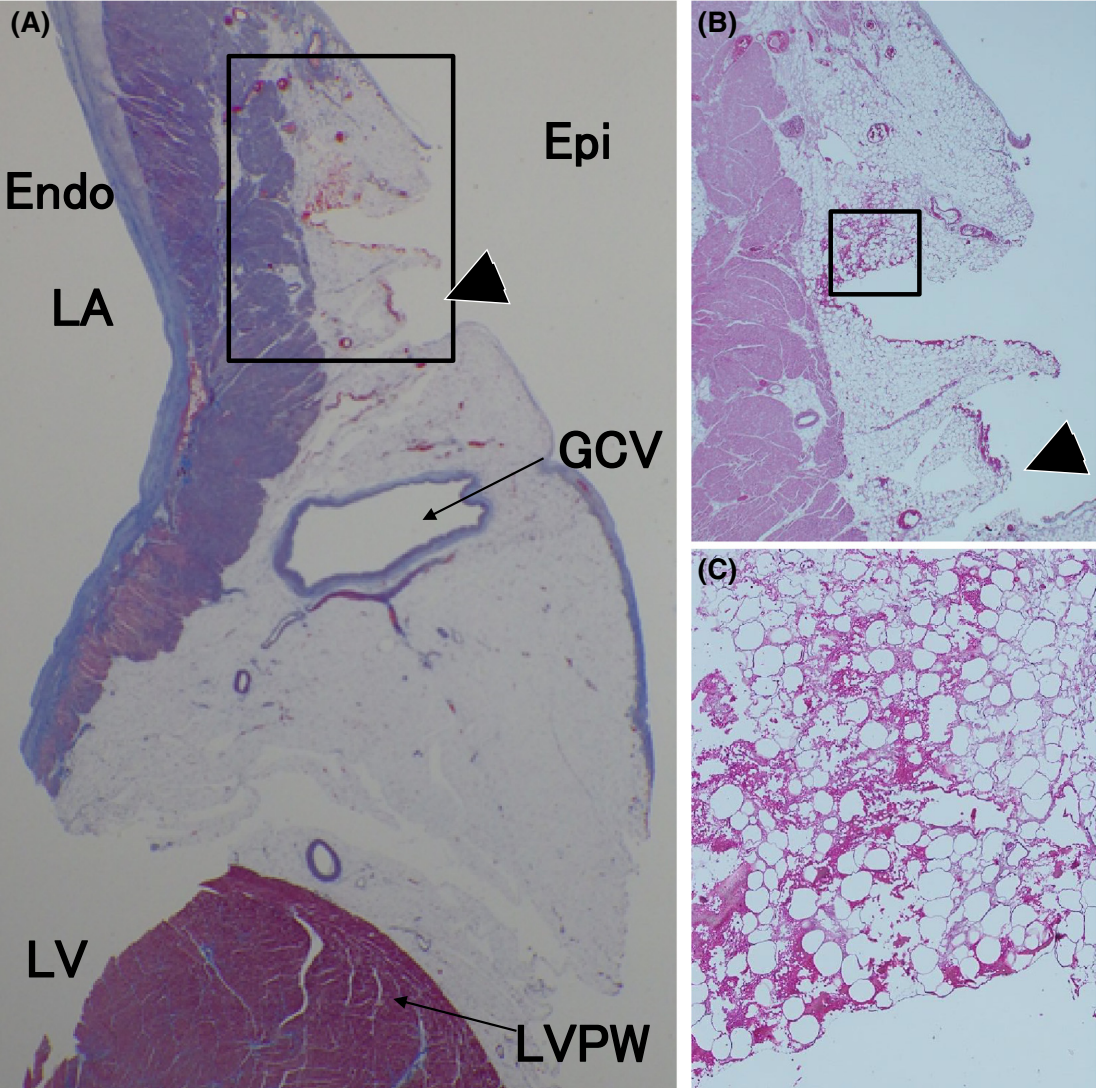
Macroscopic (A) and microscopic (B, C) examinations of the heart. (A) A Masson trichrome‐stained cross section just at the site of the laceration indicated by the dashed lines in Figure 3. (B) Magnification of the rectangular area in A (hematoxylin and eosin stain, ×20). (C) Magnification of the rectangular area in B (hematoxylin and eosin stain, ×100). The slit (black arrowhead) was created accidentally. The details are described in the text.

**Figure 5:**
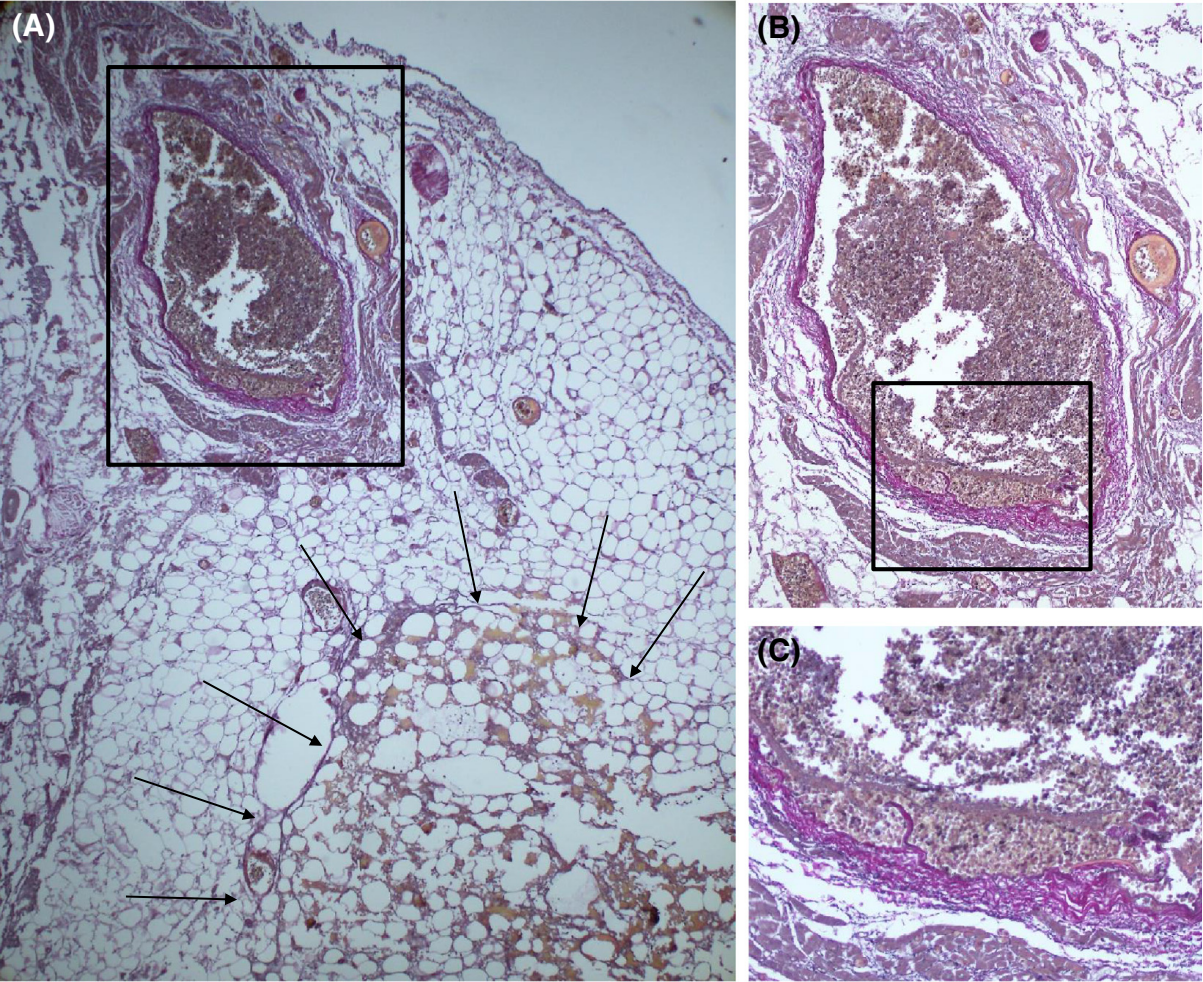
Elastica van Gieson‐stained specimens of the adjacent block of that in Figure 4. (A) The vein close to the bleeding area (×40). (B) Magnification of the rectangular area in A (×100). (C) Magnification of the rectangular area in B (×200). The details are described in the text.

A postmortem examination demonstrated ischemic necrosis of the small and large intestines, liver, and spleen, which was compatible with the CT images. The proximal mesenteric trunk was patent with no signs of a thrombotic or embolic arterial obstruction. These findings were compatible with the occurrence of NOMI. An examination of her heart revealed no injuries of any part of her heart, including the coronary sinus, other than the VOM with lacerations on the ridge of the left atrial appendage (LAA) and macroscopic clots (Fig. 3). A cross section of the histopathological specimen revealed the destruction of the vascular structure with bleeding and clots scattered inside. A microscopic examination around the lacerated VOM showed myocardial necrosis and interstitial bleeding in the adipose tissue (Fig. 4). Furthermore, in the adjacent block of that lesion, the vein wall had become thin and partial destruction, including cracks of its elastic fibers, had occurred close to the area of bleeding, suggesting the ethanol injection might have compressed the vein wall, causing the wall to rupture (Fig. 5). There were several transmural lesions created by the RF ablation applications, but there were no injuries that might have induced the pericardial effusion other than the lacerations of the VOM.

## Discussion

Chemical ablation using an ethanol injection through a vessel has been widely accepted clinically and also applied to cardiac diseases 7. Several papers have reported that ventricular arrhythmias could be treated by injecting ethanol into their culprit coronary arteries 8, 9. The complications that have been reported have been mainly caused by the reflux of the ethanol into the adjacent vessels provoking either temporary or permanent atrioventricular block or unexpected myocardial injury 8, 9. Nevertheless, they finally concluded that chemical ablation was comparatively effective and safe 8, 9. Since 2009, the EIM has been reported to be an alternative ablation method in patients with left atrial arrhythmias including atrial fibrillation 1. There have also been no serious complications regarding the EIM when used with those arrhythmias, so it was concluded that an EIM might also be safe and feasible to treat those arrhythmias 1, 4, 5, 6.

However, we experienced a patient who died of NOMI caused by transient hypotension after an EIM. An injection of ethanol and contrast medium lacerated the VOM, and did not cause a decrease in her blood pressure for some time, but eventually exacerbated her hemodynamic status following an ordinary cardioversion. Her ejection fraction was not that bad, but she could have had background heart failure with a preserved ejection fraction (HFpEF), which is common in dialysis patients 10. AF with a rapid ventricular response together with a slight pericardial effusion could have caused her collapse with HFpEF.

The time series of her hemodynamic condition suggested that the VOM that ruptured during the ethanol injection might have temporarily healed and ameliorated once, but the cardioversion tore away the thrombi that had formed leading to bleeding causing the pericardial effusion, which was compatible with the findings of the autopsy that there were no injuries except for the VOM, which was firmly covered with clots. A histopathological specimen revealed the destruction of the vascular structure with myocardial necrosis and interstitial bleeding.

One possible mechanism of the pericardial effusion might have been due to the trivial vessel injury that this patient experienced while under an inappropriate heparinized condition. However, her activated clotting time (ACT) was kept at approximately 300 sec all through the procedure, which meant her pericardial effusion developed even while her ACT was optimally controlled. She had relied on hemodialysis for more than 20 years, so her physical condition, including her heart, might have been too fragile. A conventional ACT level might have been excessive so that she had to control her heparinized state. Moreover, hemodialysis is one of the risk factors for NOMI 11, 12. Even a trivial injury could have seriously affected her hemodynamic status, and her intolerance to brief hypotension had provoked the NOMI leading to her death.

Most papers regarding EIM note that 1 mL of ethanol is injected for up to four times into the human VOM 3, 5, 6. Furthermore, Valderra′bano et al. 1 reported that they injected 5 mL of ethanol into a dog's VOM. None of those papers reported any EIM‐related complications; however, although we injected 2 mL of ethanol with each EIM into the VOM, we believe the third EIM tore the VOM. A smaller amount or fewer injections into the VOM might not have lacerated the vessel. Furthermore, earlier treatment measures for her condition might have saved her life. We should keep in mind that EIMs may not always be safe and can provoke lethal complications if the patient is fragile.

## Authorship

KK: performed conception and design, and wrote the manuscript. OI, SM, and HH: analyzed the histopathological finding. RK and AT: collected the data.

## Conflict of Interest

None declared.
